# Quantifying and predicting *Drosophila* larvae crawling phenotypes

**DOI:** 10.1038/srep27972

**Published:** 2016-06-21

**Authors:** Maximilian N. Günther, Guilherme Nettesheim, George T. Shubeita

**Affiliations:** 1 Center for Nonlinear Dynamics and Department of Physics, The University of Texas at Austin, Austin, TX 78712, USA; 2 New York University Abu Dhabi, P. O. Box 129188, Abu Dhabi, United Arab Emirates

## Abstract

The fruit fly *Drosophila melanogaster* is a widely used model for cell biology, development, disease, and neuroscience. The fly’s power as a genetic model for disease and neuroscience can be augmented by a quantitative description of its behavior. Here we show that we can accurately account for the complex and unique crawling patterns exhibited by individual *Drosophila* larvae using a small set of four parameters obtained from the trajectories of a few crawling larvae. The values of these parameters change for larvae from different genetic mutants, as we demonstrate for fly models of Alzheimer’s disease and the Fragile X syndrome, allowing applications such as genetic or drug screens. Using the quantitative model of larval crawling developed here we use the mutant-specific parameters to robustly simulate larval crawling, which allows estimating the feasibility of laborious experimental assays and aids in their design.

The broad homology between human disease genes and their counterparts in *Drosophila*^1^, as well as the extensive genetic toolbox available for the fly have led to its widespread use in studies of neurologic disorders and neural degeneration^2^,^3^,^4^. Examples include Parkinson’s and Alzheimer’s disease as well as the Fragile X syndrome. Due to the close relation between neuronal disorders and the musculoskeletal system, locomotive impairment and behavioral changes can be used diagnostically for genetic screening and characterization of disease models as well as drug screens.

While experimental methods of varying sophistication have been developed to study adult *Drosophila* behavior^5^,^6^,^7^,^8^, the altered expression of a gene very often leads to lethality at the pupal or adult stages and necessitates characterization of the larvae instead. Furthermore, the complex behaviors displayed by the larvae despite their relatively small number of 3000 functional neurons facilitate studying the neuronal basis of behaviors such as taxis, memory, learning and even social interactions^9^,^10^,^11^,^12^,^13^,^14^,^15^,^16^,^17^,^18^,^19^,^20^. Behavioral assays that describe the crawling of the larvae^3^,^11^,^12^,^13^,^14^,^15^,^16^,^17^,^20^ are typically low throughput ad-hoc assays, or assays requiring specialized equipment to follow individual larva shape changes in detail. Often, experimental geometries conceivably distort larval behavior, such as when larvae are constrained to move in a one dimensional channel. Further, manually set thresholds are frequently used to compare two individuals or populations. The ad-hoc nature of these experiments makes it difficult to compare the change in behavior under different environmental or genetic conditions and across different studies.

On the other hand, statistical analysis of larval crawling can yield a high throughput comparison between larval models of disease^21^. However, use of statistical methods alone is inherently limiting since their sensitivity depends on the quality and quantity of the collected data which could bias the description of the behavior or even miss important aspects of the phenotype. There is therefore a need for a mathematical model that circumvents these limitations to describe the crawling of larvae and additionally provide quantitative parameters that can be universally compared for various mutants or under altered environments. Previous analysis showed that larvae crawl in a superdiffusive pattern^21^, consistent with the behavior of a large set of foraging animals ranging from bumble bees to sharks^22^,^23^,^24^,^25^,^26^,^27^,^28^. Lévy flights^29^ have been used to model these behaviors, but improved analysis methods subsequently showed that this model, while consistent with some behaviors^23^,^27^,^28^, is not adequate in many cases^30^,^31^,^32^.

Here, we also show that a Lévy flight model cannot explain the crawling of the larvae. Rather, larvae trajectories can be very well described by a bimodal persistent random walk (bPRW) model. The model enables us to quantitatively describe the crawling using a small set of four parameters. We demonstrate the power of the method by comparing the crawling parameters for various genetic backgrounds, including models of neurodegenerative disease. We find that details of the crawling that were previously missed are readily captured using our method. Moreover, we show that starting from the analysis of a small sample of crawling larvae we can robustly simulate the mutant-specific crawling, reproducing the qualitative and quantitative aspects of the experimental trajectories. Using these simulations, predictions can be made on the feasibility of experiments that may require an impractically large number of individuals to reach statistical significance, and the outcome of laborious experiments can be pre-estimated by simulations to aid in their design.

## Results

### Larvae exhibit persistence and two modes of crawling

**Figure 1:**
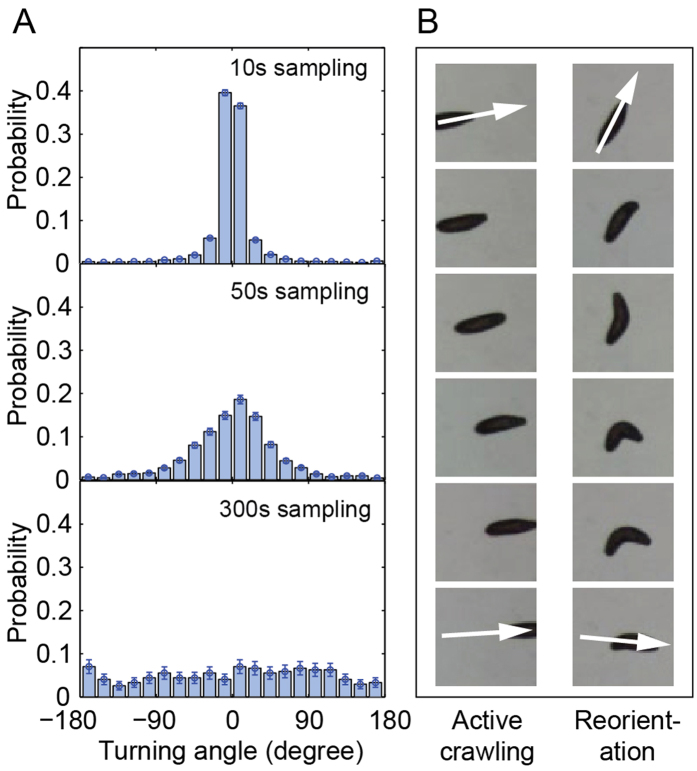
Larvae exhibit a persistent and bimodal crawling behavior. (**A**) Distributions of turning angles between consecutive segments of discrete time sampled tracks. The persistence information is gradually lost as the sampling interval is increased from 10 s to 300 s, leading to an equal distribution of turning angles, characteristic of a Brownian motion. Tracks of 25 *Drosophila* larvae are used to calculate the distributions. (**B**) The larvae exhibit two distinct walking phases: an active crawling phase (left panel), and a reorientation phase (right panel). Note the drastic angular change between the initial larval crawling path at the beginning of the reorientation phase and its end. The time lapse shows a total of 20 seconds of larval crawling in a fixed field of view of approximately 2 × 2 mm^2^.

Larvae trajectories for various strains were imaged at low magnification to record multiple larvae simultaneously and the trajectories were determined by centroid tracking (see Methods). Previous analysis of these trajectories showed that the larvae exhibit a superdiffusive crawling pattern^21^. However, Lévy flight models, previously used to explain the foraging behavior of animals^22^,^23^,^24^,^25^,^26^,^27^,^28^, do not adequately describe larval behavior (see Supplementary Material, Supplementary Table 1 and Supplementary Fig. 1). We consequently turned our search towards another model consistent with superdiffusive motion, and tested for persistent random walks (PRW)^33^. In the PRW model, the direction of a step is most likely to follow the direction of the previous step. However, the influence of the initial direction of motion vanishes over longer times^34^. Hence, the persistence information is lost when the sampling interval is large, leading to a PRW becoming a Brownian motion in the limit of large scales. Indeed, the peaked distribution of turning angles between sampling points in our experimental data suggests the involvement of persistent trajectories in the larval crawling (Fig. 1A). Consistent with theory, when the sampling interval is increased we observe loss of persistence and a convergence to Brownian motion where the probability is equal for all turning angles.

The standard PRW on its own, however, is insufficient as the larvae follow a bimodal pattern^35^, whose phases we refer to as “active crawling” and “reorientation” in the following (Fig. 1B), analogous to what was reported for other systems^36^,^37^. In the active crawling phase, a larva follows a relatively straight path, a persistent trajectory. In contrast, during the reorientation phase a larva remains at the same spot while bending and moving its head. As a result, the reorientation phase is often followed by a significant turning event. In the absence of any taxis, as in the case of our experiments, this turning is expected to be unbiased and randomly directed.

### Separating the two crawling phases in larvae trajectories

The bimodal behavior is intrinsic to the trajectory of the larvae, but comes with an inherent challenge for the observer. If a sampling interval is chosen which exceeds the average reorientation time, the reorientation events are washed-out. A lower sampling time, on the other hand, would capture the details of the head sweeping and reshaping of the larvae during the reorientation phase. These large shape changes could be misinterpreted as actual crawling by centroid tracking and add noise to trajectory analyses. This was often not accounted for^38^ and is likely to influence the qualitative conclusions of previous studies. Considering this, however, it is advantageous to record the crawling at a high enough frame rate to capture the reorientation events, and establish a means to identify these events.

Based on this idea, we developed a method that allows identifying and extracting the exact spatial and temporal distribution of the reorientation events in the tracks while preserving the advantages of our experimental setup. In particular, we do not need to rely on specialized equipment or complex and time-consuming methods, such as shape analyses or fluorescent marking, to identify the reorientation events. This in turn allows the generality and high-throughput necessary for phenotyping in relation to genetic and drug screens.

In the first step, we smooth the track by following the trajectory recorded at two second intervals and merging all points that are closer to each other than the resolution limit determined by the magnification of the imaging system and the size of the larva (see Methods). This smoothing process eliminates the underlying statistical fluctuations in the recorded tracks while preserving the hidden bimodal pattern covering large turning angles. The number of points averaged into one smoothed point, m, is saved and encodes the temporal information. Each smoothed point is consequently described by three values, its x and y positions, and m. This data set is the basis for all further analyses in the following.

**Figure 2:**
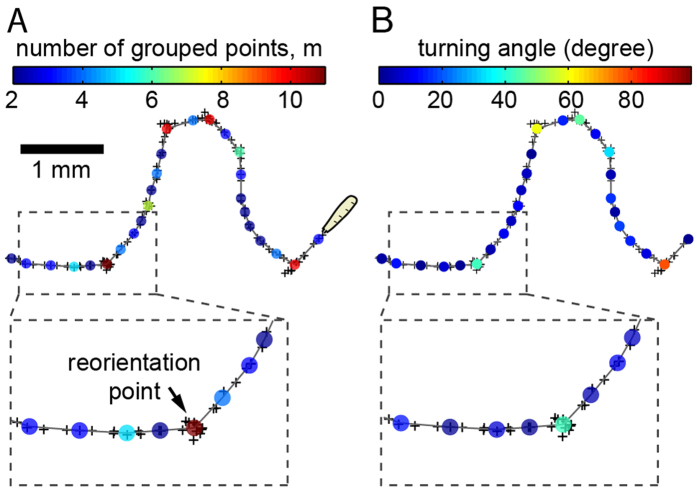
Smoothing the tracks allows identifying reorientation events. (**A**) Consecutive points (+, plus) on the trajectory recorded at discrete time intervals are grouped into one smoothed point (•, circle) if they lie within the resolution limit (see Methods). The color coding represents the number of grouped points, m. Reorientation, is typically characterized by an accumulation of sampled points (inset). A larva is drawn schematically to scale. (**B**) The smoothed points are color-coded according to the turning angle between consecutive segments. Drastic directional changes in the track are generally related to clustering of sampling points, i.e. reorientation events (inset).

Sharp edges in the smoothed tracks, related to large turning angles, are generally points with a markedly higher value of m (Fig. 2). This is consistent with the reorientation phase being a significantly slower or even still-standing phase, dominated by bending and head movement of the larva (Fig. 1B). Therefore, the m values can be used to identify reorientation positions.

**Figure 3:**
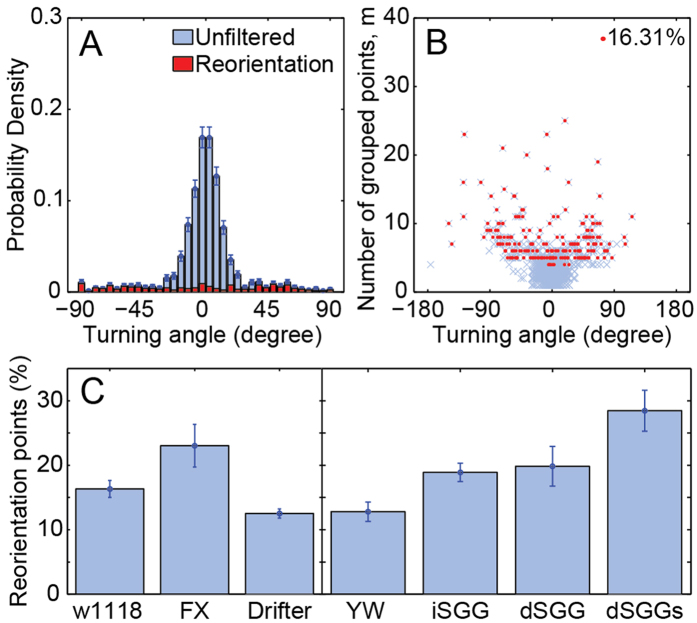
Identifying the reorientation events and comparing their frequency among mutants. (**A**) The reorientation events account for the broad underlying distribution of turning angles in the probability density distribution of turning angles between smoothed points (blue). Overlying red bars show the values identified as reorientation points, clearly covering most large turns. (**B**) Large turning angles mainly appear in reorientation events (red dots), identified by large values of the number of smoothed points (m). Blue crosses represent the unclassified data. (**C**) The frequency of reorientation events is altered in larvae mutant strains of the Fragile X syndrome model (left) and Alzheimer’s disease model (right) compared to their respective wild type strains, w1118 and YW. FX: Fragile X; iSGG: increased SGG; dSGG: decreased SGG; dSGGs: severely decreased SGG (see Methods). The percentage is calculated for smoothed points.

Given that different mutants can display different crawling speeds and reorientation characteristics (see below), fixed thresholds for mvalues cannot be used to identify reorientation. Rather, we used statistical methods that identify reorientation points as outliers in individual tracks (Thompson’s tau method^39^, see Methods). The distribution of angles between smoothed points shows that removing the reorientation points identified by this method results in an underlying level that accounts for almost all large angles being removed (Fig. 3A). This confirms the expected correlation between the clustering of smoothed points (large m) and the occurrence of a reorientation event (large turning angles) (Fig. 3B).

Thus, starting from larvae trajectories recorded at a constant rate and small spatial resolution, we are able to separate the reorientation events from the active crawling periods. This allows us to analyze the spatio-temporal distribution of these two phases and the dynamics within each phase.

### The active crawling phase is a persistent random walk

The active crawling phase is well described by a PRW as evidenced by the turning angle and step length distributions (Supplementary Tables 2–4 and Supplementary Fig. 2). The length of a step between consecutive smoothed points is well described by a Weibull distribution, while the angle between consecutive steps is normally distributed – both distributions often used to model PRWs^38^,^40^,^41^,^42^,^43^. The Weibull and normal distributions were weighted against several other distributions with maximum likelihood methods and were consistently found to be the best models for all larva strains tested (Supplementary Tables 2 and 4).

A standard PRW is fully described by its persistence length, *l*_*p*_, which can be determined by analyzing the end-to-end distance of the tracks using a method originating from polymer physics^44^. For the larval trajectories, however, the active crawling periods are interrupted by reorientation events, making the experimental segments too short to determine *l*_*p*_ reliably. Instead, we used the parameters of the step length and turning angle fits to simulate and analyze tracks consisting only of the active crawling phase (see Methods, Supplementary Table 4 and Supplementary Fig. 2). We find that the persistence length of the active crawling phase ranging 8-11 mm (Supplementary Table 5) exceeds the typical run lengths between reorientation events (4–6 mm) for all larvae strains tested (data not shown).

Given that the persistence of a trajectory determines its overall shape, we asked whether the persistence length can account for the differences in the mean squared displacement (MSD) we previously found for larvae coming from various strains^21^. We studied two disease models: a model of the Fragile X syndrome and another of Alzheimer’s disease. Larvae with reduced expression of the *Drosophila* Fragile X-related gene *dfmr1* (FX) were compared to wild type (w1118). It was shown that reduced expression of *dfmr1* in these larvae leads to increased expression of the degenerin/epithelial neuron sodium channel (DEG/ENaC) family protein Pickpocket1 (PPK1) compared to the wild type^20^. We therefore analyzed the crawling of a third strain with reduced PPK1 expression (Drifter). For the model of Alzheimer’s disease, strains with varying expression levels of the *Drosophila* homologue of Glycogen Synthase Kinase-3 (GSK-3), Shaggy (SGG), were used (see Methods)^21^,^45^ and compared to the relevant wild type (YW). Unlike the MSDs which are distinct for each strain, we find that the persistence length is comparable for most strains within a group and that the small changes do not correlate with the trends observed in the MSD (Supplementary Fig. 3). The fact that the persistence length is not a key factor of the overall larval trajectory is likely a manifestation of it being longer than the active crawling segments in between reorientation episodes (shaded range in Supplementary Fig. 3). The overall crawling behavior of the various strains must consequently lie in the spatio-temporal distribution of these two phases which we chose to investigate next.

### Differentiating mutants using their crawling parameters

To compare the crawling of the various strains we quantified the following parameters: the frequencies of reorientation events, the agility during the crawling phase, and the agility during reorientation. Reorientations are more frequent for strains with a smaller MSD (Fig. 3C), confirming that interruptions of the crawling phase dominate in determining the shape of the trajectory.

**Figure 4:**
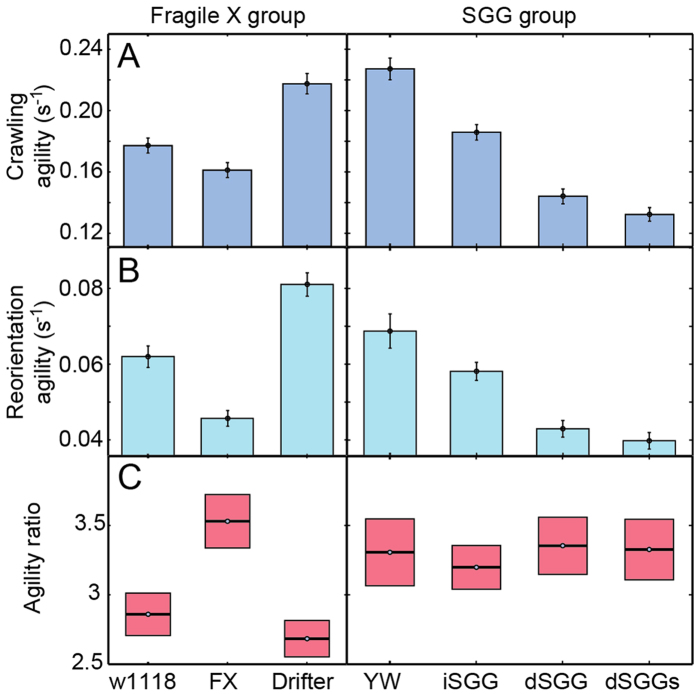
Mutation can either alter the agility of the larvae in the active crawling phase, or in the reorientation phase, or both. The inverse of the mean time in seconds spent per point (agility) quantifies the speed during the crawling phase as well as the time spent during reorientation. The agility has the same trend of change in both phases (**A,B**) for the SGG group but not the Fragile X group. This leads to the ratio of the agilities being comparable for most strains within each group (**C**). Only the Fragile X mutant stands out suggesting that the mutation primarily targets a process responsible for reorientation. Shown values are the mean and standard errors of the mean (see Supplementary Table 5).

To quantify the speed during the crawling phase as well as the time spent during reorientation, we define the agility in each phase as the inverse of the average time spent per smoothed point (Fig. 2). For all mutants in the Alzheimer’s model, where expression levels of the *Drosophila* GSK-3 homologue, SGG, are altered, both the crawling agility and the reorientation agility decrease compared to wild type (Fig. 4A,B and Supplementary Table 5). It is noteworthy that both increased and decreased expression of SGG result in reduced agility. This is consistent with our previous reports of comparable MSD for crawling larvae from both strains^21^, as well as the increased accumulations of axonal cargo in larval segmental nerves of both strains^45^. However, the severity of the agility phenotype seems to saturate upon a moderate reduction of SGG as little further impairment is observed for further reduction of SGG expression. For all mutants in the Alzheimer’s model, the ratio of the crawling and reorientation agilities is comparable (Fig. 4C, Supplementary Table 5), suggesting that mutation targets a process affecting both phases.

The Fragile X group shows a different trend. While the ratio of agilities is comparable for the wild type and Drifter strains, the Fragile X mutant has a significantly higher ratio. In particular, its agility in the active crawling phase is only slightly reduced compared to that of the wild type, but significantly reduced in the reorientation phase. For this strain, the mutation appears to primarily target a process responsible for reorientation.

### Statistical analyses of simulated and measured larvae trajectories converge

**Figure 5:**
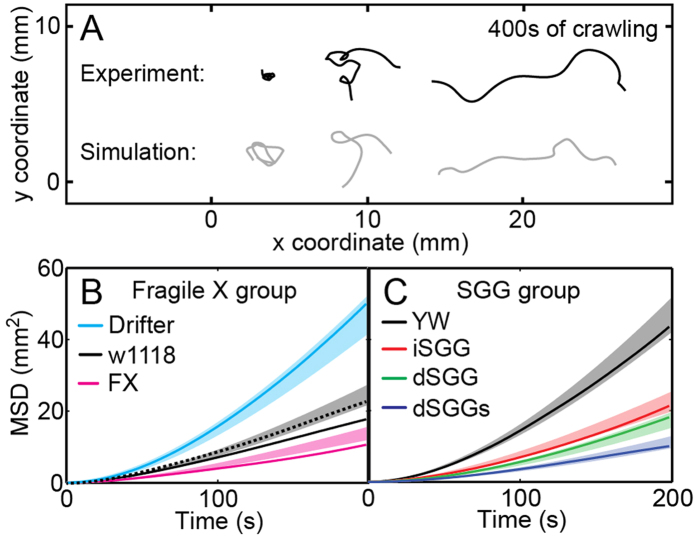
Experimental and simulated larvae trajectories converge. (**A**) Trajectories simulated using the crawling parameters of one strain cover the whole variety of morphologies observed experimentally within this strain. The variation is intrinsic in our mathematical model, suggesting that the observed experimental variety is not conditioned by individual defects. (**B**) Most strains show experimentally measured MSDs (solid lines) that lie within the simulated one standard deviation (1*σ*) band (colored areas); exceptions are the w1118 wild type and Fragile X mutant. However, a repeated control experiment for the w1118 strain (dashed black line) lies within the 1*σ* band indicating that the deviation observed is due to statistical variation which is intrinsic in the definition of the standard deviation encompassing 68% of the data. The *σ* bands of the MSD were calculated by running 1000 simulations each with trajectories equal in number to the experimental trajectories for each strain. The control experiment is based on 14 larvae. All other experimental trajectories are the same ones we studied previously (see [Jakubowski 2012] and Methods).

We next asked whether the parameters quantified above to describe the various mutants can reproduce the overall trajectory morphology which we previously quantified using the MSD and showed is sensitive to mutation^21^. We therefore generated simulated larvae trajectories with the mutant-specific parameters as input (see Methods, Supplementary Tables 5 and 6). Indeed, simulated trajectories appeared similar to measured trajectories of crawling larvae, covering the full variety of the track morphologies (Fig. 5A). Further, the probability distributions and crawling parameters extracted from simulated trajectories matched those of the experiment as anticipated.

As expected for a limited sample size of trajectories, the MSD varied, but different mutants were indeed distinct (Fig. 5B). The measured MSDs for most mutants fell within one standard deviation (1σ) of the simulated trajectories, with the exception of two out of the seven strains. This could either be indicative of our model missing an aspect of the crawling that is important for these mutants, or just a manifestation of statistical variation. To rule out the former possibility, another data set of crawling larvae was recorded and tracked for the w1118 strain which lies furthest from the 1σ band. The MSD for the new data set lies well within the one standard deviation band suggesting that the deviation of the original data set is statistical (Fig. 5B). Significantly, the crawling parameters extracted from the new data set coincide with those of the original data set used to generate the simulated tracks (Supplementary Table 5).

Finally, we sought an independent method to test whether the simulated and experimental trajectories belong to the same class of random walks as they should if the simulation captures the larval crawling. We therefore used a recently reported methodology which uses renormalization group to classify diffusion processes^46^,^47^. This method has been shown to differentiate between stochastic processes which have indistinguishable MSD. We find that experimental trajectories for all strains fall in the same class of random walks and that experiments and simulations agree (Supplementary Fig. 4). These findings lead us to conclude that our model indeed captures the larval crawling which enables us to get more insight into the details of the crawling than statistical measures alone provide.

## Discussion

The crawling of *Drosophila* larvae can be well described by a bimodal persistent random walk (bPRW) model that accounts for the two phases of the crawling: active crawling and reorientation episodes. The analysis method we developed uses as input the trajectory of larvae that can be recorded using a simple camera and lens apparatus^21^. Higher magnification imaging, tracking and body-shape-change recording is possible^13^,^14^,^16^,^20^ and can be important to understand crawling mechanisms. However, the simplicity of the present method, coupled with the model-based analysis, allows the high throughput required for screens without compromising the sensitivity. This is illustrated by our analysis of the seven different larval strains, including models of neuronal disorders.

Upon comparing the parameters that quantify the crawling of the various larvae strains two themes emerge. First, mutants have distinct crawling characteristics that can be used to differentiate between them. Thus, the high-throughput, sensitive, and robust nature of the method make it suitable for large genetic screens or drug screens using larval models of disease. While we demonstrated the capability of the method using models of neuronal disorders, the method is more general and can be readily used in relation to other diseases or for studies of behavioral change. This includes the response to environmental cues such as studies of neuronal networks involved in vision, olfaction, gustation, learning or social interaction.

Second, the parameters of the crawling altered by mutation can help infer insight into which neuronal pathways used for crawling are targeted. Recent work has shown that larval crawling can be described by two motor programs. Peristalsis drives forward movement, while asymmetric contraction of the anterior body segments of larvae drives head sweeping movement^14^. It can be tempting to map these two motor programs to the two phases of crawling in our model: active crawling and reorientation, respectively. However, peristalsis was shown to be important both for the forward movement (active crawling) as well as for exiting the reorientation events^14^. With this in mind, we can relate the altered agilities in the two phases for the various strains (Fig. 4) to these two motor programs. For most mutants within a disease model, the ratio of the active crawling to reorientation agilities does not change (Fig. 4C) suggesting that the two motor programs important for both phases are targeted by the mutation. The Fragile X mutant stands out since its reorientation agility is reduced more pronouncedly than the crawling agility, suggesting that the mutation primarily targets the asymmetric contractions of the anterior body segments. Intriguingly, comparison of the agility patterns of the Fragile X and Drifter mutants (Fig. 4) lead to the conclusion that the molecular basis of these mutations target neuronal circuits that control distinct motor programs. This insight is completely missed in model-free statistical analyses of the crawling of these two mutants which simply show impaired crawling for the Fragile X and enhanced crawling for the Drifter strain compared to wild-type^20^,^21^. This alteration in the behavior was attributed to the reduction of PPK1 expression in the Drifter mutants and its over-expression in the Fragile X mutant due to the loss-of-function of DFMR1 which inhibits PPK1 expression in wild-type larvae^20^. However, our analysis strongly suggests that PPK1 expression is not the sole determinant of the crawling behavior that is targeted in the Fragile X mutant. While future work will be needed to identify the target, our finding illustrates that hypotheses about the neural basis of mutant behavior can be formed using the analysis of the present methodology.

Trajectories simulated using the parameters of the bPRW model have average morphologies that quantitatively agree with those of experimental trajectories for each strain (Fig. 5) and, together with the experimental trajectories, globally belong to the same class of diffusion processes (Supplementary Fig. 4). In addition to demonstrating the validity of the model, simulated trajectories can be used to design experiments or estimate their outcome. In particular, the number of individuals needed for an experiment to reach statistical significance can be determined by simulation. As the MSD analysis in Fig. 5 demonstrates, the wide variability of the individual trajectories from each strain can lead to under-sampling. Experiments that involve counting the number of larvae crawling past a threshold are common in behavioral or taxis studies, and could benefit from simulations when designing the experiments.

## Materials and Methods

### Fly stocks, apparatus design and tracking

Larval crawling trajectories were recorded as previously described^21^. Briefly, flies were grown in cups on standard agar-yeast media and multiple late embryos were collected and placed on a 15 cm petri dish filled uniformly with agar-apple juice media. A simple camera and lens system on a stand was used to record low magnification time-lapse images of the crawling larvae at a rate of one frame every two seconds once the embryos hatched. Trajectories were digitized using a previously-described centroid-based tracking algorithm^48^. All stocks were obtained from the Bloomington Drosophila Stock Center, unless otherwise specified. Wild type controls were either w1118 (FBid: 6326) or YW. The *dfmr1*^4^ stock we used as our Fragile X model (FX) has been described previously^49^,^50^ and was provided to us by Fen-Biao Gao (UCSF). The Drifter (Df) mutation was created by crossing Gal4 109(2)80 (FBid: 8768) virgins to UAS-ppk1-RNAi (TRiP, Harvard Medical School, FBid: 29571 ) males, achieving over-expression of ppk1-RNAi in all multiple dendritic neurons. This stock has been described extensively^51^. Over-expression of dGSK-3 (iSGG in main test) was accomplished by crossing elav Gal4 (FBid: 458) virgins with UAS-SggS9A (FBid: 5255) males. For dGSK-3 reduction *sgg*^1^*/FM7, Actin-GFP* females were crossed to *sgg*^1^*/Dp(1;2;Y)w*^*+*^ (FBid: 4095) males that survive due to a small X duplication containing the *Sgg* gene on their Y chromosome^45^. The first-instar larvae expressing GFP as seen under a dissection fluorescence microscope were termed the ‘dSGG’ (decreased SGG) population while the GFP-negative larvae were termed the ‘dSGGs’ (severly decreased SGG) population, based on the corresponding dGSK-3 expression levels. The same data set reported previously^21^ was used in the current analysis. Exceptions are additional trajectories collected for the Fragile X wild type, w1118 (see Fig. 5).

### Data analysis with the MaggMa software package

All data used in this work, as well as the MATLAB scripts used in our MaggMa analysis software package, will be available online or can be requested from the corresponding author.

Discrete-time sampled coordinates of the animal’s centroid generated by the tracking algorithm are used as the input for the analysis. The time resolution should be high enough to ensure a sufficient amount of sampling points within an average reorientation event. We chose to capture an image every two seconds based on the nominal crawling speeds of the larvae of around one body length every 15 seconds.

The recorded trajectories were first smoothed by following the trajectory and summarizing all points that are closer to each other than a threshold *r*. The threshold, or resolution limit *r*, was chosen empirically to be the nominal width of a larva as it appears in camera image. The algorithm starts from the first sampling point 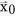 and measures the spatial distance d to the consecutive sampling point 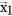 as 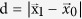. If d is smaller than or equal to *r*, it is redefined as the distance between 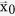 and the next consecutive sampling point 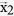. This procedure is repeated until the first sampling point 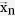 for which 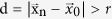 is reached. The sequence of sampling points 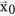 to 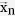 is used to calculate a smoothed point 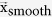 by


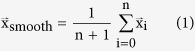


The consecutive sampling point 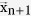 is then reset as 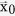 and the procedure is repeated until the end of the track is reached. To ensure the finest possible smoothing, we improved this method by setting the threshold to *a·r*, where *a* is a scaling factor increasing from 0.1 to 1 in steps of 0.1 after each complete run of the algorithm. At the end of this procedure, the number of points averaged into one smoothed point, m = n + 1, is saved, so that any smoothed point is described by three values, its x and y position, and m. This data set was used as the basis for all further analysis in the current work.

In the second step, we analyze the m values of each single track to identify the largest values, which then are identified as reorientation points. For this purpose we applied Thompson’s tau method^39^, which can be thought of as an adaptive two sigma outlier detection algorithm.

In very rare cases we found tracks where the larvae barely moved, possibly due to injury while placing the embryo on the agar plate. To avoid the influence of these, we have additionally filtered out all instances where the larvae rest in one point for more than 14 seconds. This threshold has been empirically set to ensure accounting for these tracks without influencing the majority. The average time per active crawling point (CP) lies in all cases significantly below 14 seconds (see Supplementary Table 5), so that misclassifications can be ruled out.

### Simulating tracks with the MaggMa software package

For the simulation of the larvae tracks we used the extracted parameters employed in the main text to compare between and differentiate the various mutant backgrounds and models of neurological disorders. The set of parameters used in the simulation consists of 1) the mean frequency of reorientation points, 2) the distributions of the time per active crawling and per reorientation point, 3) the step length between two smoothed points as well as 4) the Gaussian standard deviation of the turning angles during the active crawling phase and after reorientation events (Supplementary Tables 5 and 6).

These are a set of six parameters even though only a set of four parameters was needed to differentiate between the disease models. However, the step length and Gaussian standard deviation during the active crawling phase are both characterized by the single parameter of persistence length as described in the main text and Supplementary Fig. 2. The distributions were used in the simulation instead for convenience, but cannot be considered as two separate parameters.

The time per active crawling and reorientation point reported as average values in Supplementary Table 5 and Fig. 4 in the main text were modeled as Gamma distributions that account well for the long-tailed distributions observed for these parameters. These distributions lead to variability in the individual simulated tracks that is reminiscent of the variability observed in experiments. The statistical average on the population level converges naturally to the mean of the respective Gamma distribution, which in all cases was equal to the measured mean value within statistical errors.

The distribution of turning angles after a reorientation event was not used to differentiate mutants due to low statistics and high uncertainty. For the purpose of the simulation, however, the standard deviation of these turning angles for each mutant was used to model the observed spread. See Supplementary Table 6 for the values for each strain.

### Algorithm implementations used in the MaggMa analysis software

Identifying the reorientation events is based on Thompson’s tau method^39^. We implemented the MATLAB code ‘find_outliers_Thompson.m’ by Michele Rienzner in our algorithms for the purpose of this test statistic.

To determine the standard deviation of the broad distribution of turning angles after a reorientation event, we fit a Gaussian to the distribution using the MATLAB code ‘deleteoutliers.m’ by Brett Shoelson to filter out individual outliers with the Grubb’s test. The Grubb’s test for outlier detection is specifically adjusted for normally distributed data^52^. This was necessary since the number of data for these was strongly limited and maximum likelihood fitting would have been strongly influenced by individual outliers.

For comparison of different distributions for a data sample we used in most cases the MATLAB code ‘allfitdist’ by Mike Sheppard, which implemented maximum likelihood fitting for a multitude of the most common distributions. For fitting of the Cauchy distribution we used Peder Axensten’s MATLAB programs ‘cauchyfit.m’ and ‘paxmle.m’. We used the MATLAB scripts ‘suptitle.m’ by Drea Thomas and ‘subtightplot.m’ by Felipe G. Nievinski to generate the graphics of some of the displayed figures.

## Additional Information

**How to cite this article**: Günther, M. N. *et al*. Quantifying and predicting *Drosophila* larvae crawling phenotypes. *Sci. Rep.* **6**, 27972; doi: 10.1038/srep27972 (2016).

## Supplementary Material

Supplementary Information

## References

[b1] Reiter L. T., Potocki L., Chien S., Gribskov M. & Bier E. A systematic analysis of human disease-associated gene sequences in Drosophila melanogaster. Genome Res 11, 1114–1125, 10.1101/gr.169101 (2001). 11381037 PMC311089

[b2] Ambegaokar S. S., Roy B. & Jackson G. R. Neurodegenerative models in Drosophila: polyglutamine disorders, Parkinson disease, and amyotrophic lateral sclerosis. Neurobiol Dis 40, 29–39 (2010). 20561920 10.1016/j.nbd.2010.05.026 PMC2926295

[b3] LeDoux M. Movement Disorders: Genetics and Models. (Academic Press, 2005).

[b4] Sang T.-K. & Jackson G. R. Drosophila Models of Neurodegenerative Disease. NeuroRx 2, 438–446 (2005). 16389307 10.1602/neurorx.2.3.438 PMC1144487

[b5] Donelson N. C. . High-resolution positional tracking for long-term analysis of Drosophila sleep and locomotion using the “tracker” program. PLoS One 7, e37250, 10.1371/journal.pone.0037250 (2012). 22615954 PMC3352887

[b6] Feiguin F. . Depletion of TDP-43 affects Drosophila motoneurons terminal synapsis and locomotive behavior. FEBS Letters 583, 1586–1592 (2009). 19379745 10.1016/j.febslet.2009.04.019

[b7] Jahn T. R. . Detection of early locomotor abnormalities in a Drosophila model of Alzheimer’s disease. J Neurosci Methods 197, 186–189 (2011). 21315762 10.1016/j.jneumeth.2011.01.026 PMC3712187

[b8] Slawson J. B., Kim E. Z. & Griffith L. C. High-resolution video tracking of locomotion in adult Drosophila melanogaster. J. Vis. Exp. 24, 10.3791/109 (2009). PMC2762895 19390509

[b9] Aceves-Pina E. O. & Quinn W. G. Learning in normal and mutant Drosophila larvae. Science 206, 93–96, 10.1126/science.206.4414.93 (1979). 17812455

[b10] Durisko Z. & Dukas R. Attraction to and learning from social cues in fruitfly larvae. Proc Biol Sci 280, 20131398, 10.1098/rspb.2013.1398 (2013). 23902906 PMC3735258

[b11] Gomez-Marin A., Partoune N., Stephens G. J., Louis M. & Brembs B. Automated tracking of animal posture and movement during exploration and sensory orientation behaviors. PLoS One 7, e41642, 10.1371/journal.pone.0041642. 22912674 PMC3415430

[b12] Gomez-Marin A., Stephens G. J. & Louis M. Active sampling and decision making in Drosophila chemotaxis. Nat Commun 2, 441, 10.1038/ncomms1455 (2011). 21863008 PMC3265367

[b13] Kane E. A. . Sensorimotor structure of Drosophila larva phototaxis. Proc Natl Acad Sci USA 110, E3868–3877, 10.1073/pnas.1215295110 (2013). 24043822 PMC3791751

[b14] Lahiri S. . Two alternating motor programs drive navigation in Drosophila larva. PLoS One 6, e23180, 10.1371/journal.pone.0023180 (2011). 21858019 PMC3156121

[b15] Louis M., Huber T., Benton R., Sakmar T. P. & Vosshall L. B. Bilateral olfactory sensory input enhances chemotaxis behavior. Nat Neurosci 11, 187–199, 10.1038/nn2031 (2008). 18157126

[b16] Luo L. . Navigational decision making in Drosophila thermotaxis. J Neurosci 30, 4261–4272, 10.1523/JNEUROSCI.4090-09.2010 (2010). 20335462 PMC2871401

[b17] Mudher A. . GSK-3beta inhibition reverses axonal transport defects and behavioural phenotypes in Drosophila. Mol Psychiatry 9, 522–530 (2004). 14993907 10.1038/sj.mp.4001483

[b18] Scherer S., Stocker R. F. & Gerber B. Olfactory learning in individually assayed Drosophila larvae. Learn Mem 10, 217–225, 10.1101/lm.57903 (2003). 12773586 PMC202312

[b19] Sokolowski M. B. Foraging strategies of Drosophila melanogaster: a chromosomal analysis. Behav Genet 10, 291–302 (1980). 6783027 10.1007/BF01067774

[b20] Xu K. . The fragile X-related gene affects the crawling behavior of Drosophila larvae by regulating the mRNA level of the DEG/ENaC protein pickpocket1. Curr. Biol. 14, 1025–1034 (2004). 15202995 10.1016/j.cub.2004.05.055

[b21] Jakubowski B. R., Longoria R. A. & Shubeita G. T. A high throughput and sensitive method correlates neuronal disorder genotypes to Drosophila larvae crawling phenotypes. Fly 6, 303–308, 10.4161/fly.21582 (2012). 22992470 PMC3519666

[b22] De Jager M., Weissing F. J., Herman P. M., Nolet B. A. & van de Koppel J. Levy walks evolve through interaction between movement and environmental complexity. Science 332, 1551–1553, 10.1126/science.1201187 (2011). 21700872

[b23] Humphries N. E. . Environmental context explains Levy and Brownian movement patterns of marine predators. Nature 465, 1066–1069 (2010). 20531470 10.1038/nature09116

[b24] Sims D. W. . Scaling laws of marine predator search behaviour. Nature 451, 1098–1102, 10.1038/nature06518 (2008). 18305542

[b25] Viswanathan G. M. . Levy flight search patterns of wandering albatrosses. Nature 381, 413–415 (1996). 10.1038/nature06199 17960243

[b26] Viswanathan G. M. . Optimizing the success of random searches. Nature 401, 911–914 (1999). 10553906 10.1038/44831

[b27] Bartumeus F., Peters F., Pueyo S., Marrase C. & Catalan J. Helical Levy walks: adjusting searching statistics to resource availability in microzooplankton. Proc Natl Acad Sci USA 100, 12771–12775, 10.1073/pnas.2137243100 (2003). 14566048 PMC240693

[b28] Harris T. H. . Generalized Levy walks and the role of chemokines in migration of effector CD8+ T cells. Nature 486, 545–548, 10.1038/nature11098 (2012). 22722867 PMC3387349

[b29] Zaburdaev V., Denisov S. & Klafter J. Levy walks. Rev Mod Phys 87, 483–530, 10.1103/RevModPhys.87.483 (2015).

[b30] Edwards A. M. Overturning conclusions of Levy flight movement patterns by fishing boats and foraging animals. Ecology 92, 1247–1257 (2011). 21797153 10.1890/10-1182.1

[b31] Edwards A. M., Freeman M. P., Breed G. A. & Jonsen I. D. Incorrect likelihood methods were used to infer scaling laws of marine predator search behaviour. PloS one 7, e45174 (2012). 23071508 10.1371/journal.pone.0045174 PMC3465316

[b32] Edwards A. M. . Revisiting Levy flight search patterns of wandering albatrosses, bumblebees and deer. Nature 449, 1044–1048 (2007). 17960243 10.1038/nature06199

[b33] Patlak C. Random walk with persistence and external bias. The bulletin of mathematical biophysics 15, 311–338, 10.1007/bf02476407 (1953).

[b34] Benhamou S. How many animals really do the Levy walk? Ecology 88, 1962–1969 (2007). 17824427 10.1890/06-1769.1

[b35] Green C. H., Burnet B. & Connolly K. J. Organization and patterns of inter- and intraspecific variation in the behaviour of Drosophila larvae. Anim. Behav. 31, 282–291, doi: http://dx.doi.org/10.1016/S0003-3472(83)80198-5 (1983).

[b36] Berg H. C. & Brown D. A. Chemotaxis in Escherichia coli analysed by three-dimensional tracking. Nature 239, 500–504 (1972). 4563019 10.1038/239500a0

[b37] Potdar A. A., Lu J., Jeon J., Weaver A. M. & Cummings P. T. Bimodal analysis of mammary epithelial cell migration in two dimensions. Ann Biomed Eng 37, 230–245, 10.1007/s10439-008-9592-y (2009). 18982450 PMC3586332

[b38] Holzmann H., Munk A., Suster M. & Zucchini W. Hidden Markov models for circular and linear-circular time series. Environ Ecol Stat 13, 325–347, 10.1007/s10651-006-0015-7 (2006).

[b39] Thompson W. R. On a Criterion for the Rejection of Observations and the Distribution of the Ratio of Deviation to Sample Standard Deviation. Ann. Math. Stat. 6, 214–219 (1935).

[b40] Bartumeus F., Da Luz M. G. E., Viswanathan G. M. & Catalan J. Animal Search Strategies: A Quantitative Random-Walk Analysis. Ecology 86, 3078–3087, doi: 10.2307/3450820 (2005).

[b41] Cain M. L. Random Search by Herbivorous Insects: A Simulation Model. Ecology 66, 876–888, 10.2307/1940550 (1985).

[b42] Crist T. O. & MacMahon J. A. Individual foraging components of harvester ants: movement patterns and seed patch fidelity. Insectes Sociaux 38, 379–396 (1991).

[b43] Morales J. M., Haydon D. T., Frair J., Holsinger K. E. & Fryxell J. M. Extracting more out of relocation data: building movement models as mixtures of random walks. Ecology 85, 2436–2445, 10.1890/03-0269 (2004).

[b44] Wilhelm J. & Frey E. Radial Distribution Function of Semiflexible Polymers. Phys. Rev. Lett. 77, 2581–2584 (1996). 10061990 10.1103/PhysRevLett.77.2581

[b45] Weaver C. . Endogenous GSK-3/shaggy regulates bidirectional axonal transport of the amyloid precursor protein. Traffic 14, 295–308, 10.1111/tra.12037 (2013). 23279138

[b46] O’Malley D. & Cushman J. H. A Renormalization Group Classification of Nonstationary and/or Infinite Second Moment Diffusive Processes. J Stat Phys 146, 989–1000, 10.1007/s10955-012-0448-3 (2012). 23005387

[b47] Regner B. M., Tartakovsky D. M. & Sejnowski T. J. Identifying Transport Behavior of Single-Molecule Trajectories. Biophys J 107, 2345–2351, 10.1016/j.bpj.2014.10.005 (2014). 25418303 PMC4241458

[b48] Carter B. C., Shubeita G. T. & Gross S. P. Tracking single particles: a user-friendly quantitative evaluation. Phys Biol 2, 60–72, 10.1088/1478-3967/2/1/008 (2005). 16204858

[b49] Lee A. . Control of dendritic development by the Drosophila fragile X-related gene involves the small GTPase Rac1. Development (Cambridge, England) 130, 5543–5552 (2003). 10.1242/dev.00792 14530299

[b50] Siomi H., Siomi M. C., Nussbaum R. L. & Dreyfuss G. The protein product of the fragile X gene, FMR1, has characteristics of an RNA-binding protein. Cell 74, 291–298 (1993). 7688265 10.1016/0092-8674(93)90420-u

[b51] Ainsley J. A. . Enhanced locomotion caused by loss of the Drosophila DEG/ENaC protein Pickpocket1. Curr. Biol. 13, 1557–1563 (2003). 12956960 10.1016/s0960-9822(03)00596-7

[b52] Grubbs F. E. Procedures for Detecting Outlying Observations in Samples. Technometrics 11, 1-&, 10.2307/1266761 (1969).

