# Supplementary material for: Quantifying and predicting Drosophila larvae crawling phenotypes
Source: Sci Rep. 2016 Jun 21;6:27972. doi: 10.1038/srep27972 (PMC4914969; doi:10.1038/srep27972)
Supplement: Supplementary Information [file srep27972-s1.pdf]

Quantifying and predicting *Drosophila* larvae crawling phenotypes

Maximilian N. Günther[1], Guilherme Nettesheim[1] and George T. Shubeita[1,2]

[1] Center for Nonlinear Dynamics and Department of Physics, The University of Texas at Austin, Austin, TX 78712

[2] New York University Abu Dhabi, P. O. Box 129188, Abu Dhabi, United Arab Emirates

Supplement

Larval crawling is *not* described by a Lévy flight

Previous mean squared displacement (MSD) analysis of the larval crawling showed that the crawling pattern is super-diffusive resulting in the slope of the MSD being larger than one[1]. Lévy flights have been used in the past to describe the super-diffusive foraging behavior of various animals[2-6]. However, many of these were later shown not to be Lévy flights when tested using improved analysis methods[7-9]. We used the recent methods[10,11] to test whether the larvae perform Lévy flights (Supplementary Fig. 1). Larval trajectories were segmented into straight paths and the distribution of the lengths of these paths (flights) was analyzed. The survival probability distribution of flight lengths (1 – F(x) where F(x) is the probability density function) is fit well with an exponential decay which is very different from the power law expected for the heavy-tailed Lévy distribution. Moreover, the distribution of segment lengths after logarithmic binning and normalization (LBN) is again consistent with an exponential decay rather than the straight line expected for a Lévy distribution tail. These data strongly suggest that the crawling of the larvae cannot be described by a Lévy flight. In support of this, unlike Lévy flights which are scale invariant, the larvae trajectories show persistence, since reducing the sampling rate results in loss of persistence (Fig. 1A).

Supplementary Figures

Supplementary Figure 1

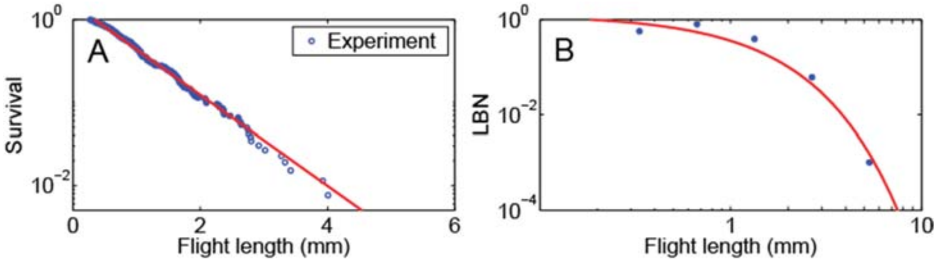

***<u>Supplementary Figure 1:</u>* Larval trajectories cannot be described by a Lévy flight.** *We used the analyses of the survival probability as well as the logarithmic binning and normalization (LBN) described by Sims et al.[11] to search for any Lévy flight pattern in the larvae trajectories. (A) Semi-logarithmic plot of the survival probability (blue dots) which describes the ratio of all flights with a flight length larger than the value at each point. The survival probability is best explained by a straight line fit (red line). This corresponds to an exponential decrease of the tail of the distribution. A heavy-tailed distribution (as for a Lévy flight) would instead follow a power law, characterized by a straight line in a double logarithmic plot of the survival probability. For the fitting the lowest as well as highest 5% of the data were excluded (blue empty circles) to avoid the influence of statistical fluctuations. (B) For the LBN plot the data is binned on a logarithmic scale with logarithmically increasing bin widths, and each bin is further normalized by the bin size (blue dots). A heavy-tailed distribution that would be consistent with a Lévy flight would follow a straight line with a slope between 1 and 3. Instead, we find a good agreement with the exponential distribution* $\lambda e^{\lambda x}$ *(red curve), whose parameter* λ *was estimated using the straight line fit to the survival probability in (A). Thus, both methods of analysis strongly suggest that the larval crawling is not consistent with Lévy flights. Data shown is for the w1118 wild type strain but similar conclusions were found for all the other strains used in this work. Fitting parameters for all strains can be found in Supplementary Table 1.*

Supplementary Figure 2

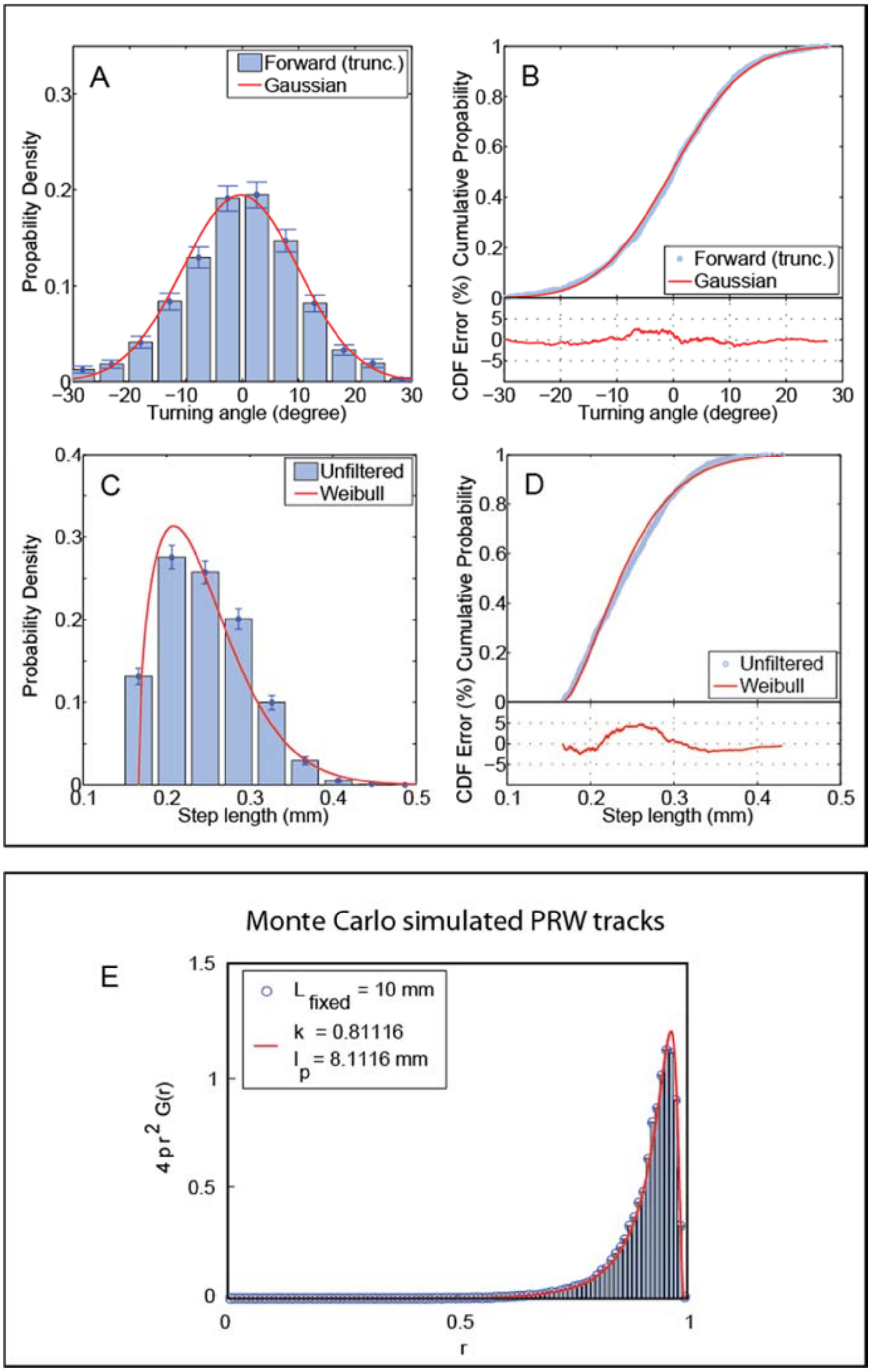

***<u>Supplementary Fig. 2:</u>* Modeling of the active crawling phase as a Persistent Random Walk (PRW).** *The turning angle distribution between consecutive points of the smoothed tracks is best fitted by a normal distribution, which was determined using Maximum Likelihood Estimation (MLE) (see Methods and Supplementary Table 4). The corresponding normal distribution (red) is graphically compared to the experimental probability density distribution (blue in (A)) and cumulative probability with error (blue in*

(B)). The error (CDF Error) was calculated as the difference between the two cumulative probability values of each point. (C) The spatial distances between successive smoothed points are best fitted by a Weibull distribution as MLE estimations have shown (see Methods and Supplementary Table 2). The distribution is shifted by 0.167mm to account for the lower limit used in the smoothing procedure described in the main text and Methods. The Weibull distribution determined using MLE (red) is compared to the experimental probability density distribution (blue in (C)) and cumulative probability with error (blue in (D)). Data shown in the figure is for the w1118 strain, but similar results were found for all other strains used in this work. The parameters for the normal distribution of turning angles and the Weibull distributions for step lengths for all strains are summarized in Supplementary Tables 4 and 3, respectively. (E) Using the parameters of Weibull and normal distribution, we simulated tracks based on the parameters of the active crawling phase for each strain (see Methods). Reorientation episodes were purposely not included in these trajectories to enable quantifying the persistence length of the active crawling phase. These tracks were analyzed by fitting the end-to-end distribution function to the measured data[12], allowing a determination of the persistence length for the crawling phase of the respective strain.

Supplementary Figure 3

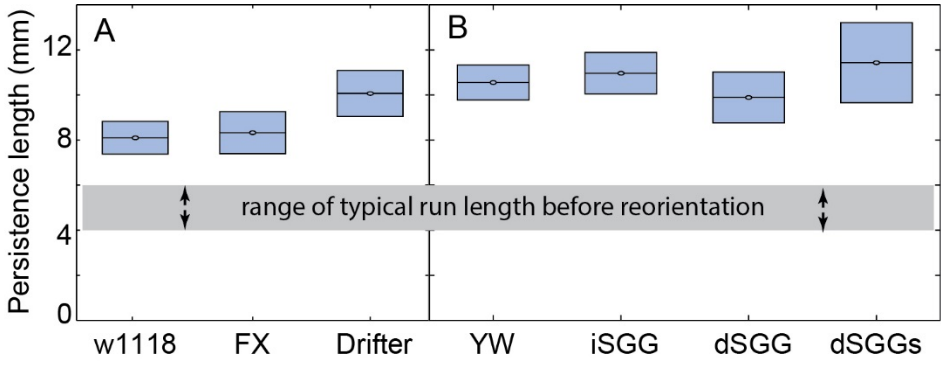

<u>Supplementary Figure 3:</u> The persistence length of the active crawling phase for the various strains. *Persistence lengths (l_p) were estimated by fitting the end-to-end distribution function for the trajectories as described in Supplementary Figure 2E. The persistence length does not change much even for larva strains that exhibit very distinct trajectory morphologies quantified by the mean squared displacement. This suggests that the persistence length is not a good descriptor of the crawling. Moreover, the value of the persistence length (8-11mm) is larger than the typical run length (4-6 mm, grey band) between consecutive reorientation events for all strains. This implies that it is the reorientation events rather than the slight bending of the persistent crawling phase that determine the overall morphology of the larval trajectory. (A) The Fragile X group: w1118 is the wild type strain, FX mutant larvae express reduced amounts of the Fragile X-related gene dfmr1, and Drifter larvae have reduced expression of the channel subunit PPK1. (B) The SGG group model for Alzheimer disease: YW is the wild type strain, with the GSK-3 homologue SGG expression levels increased for iSGG, decreased for dSGG, and more severely decreased for the dSGGs strain (Methods). The values of l_p for all strains are summarized in Supplementary Table 5.*

Supplementary Figure 4

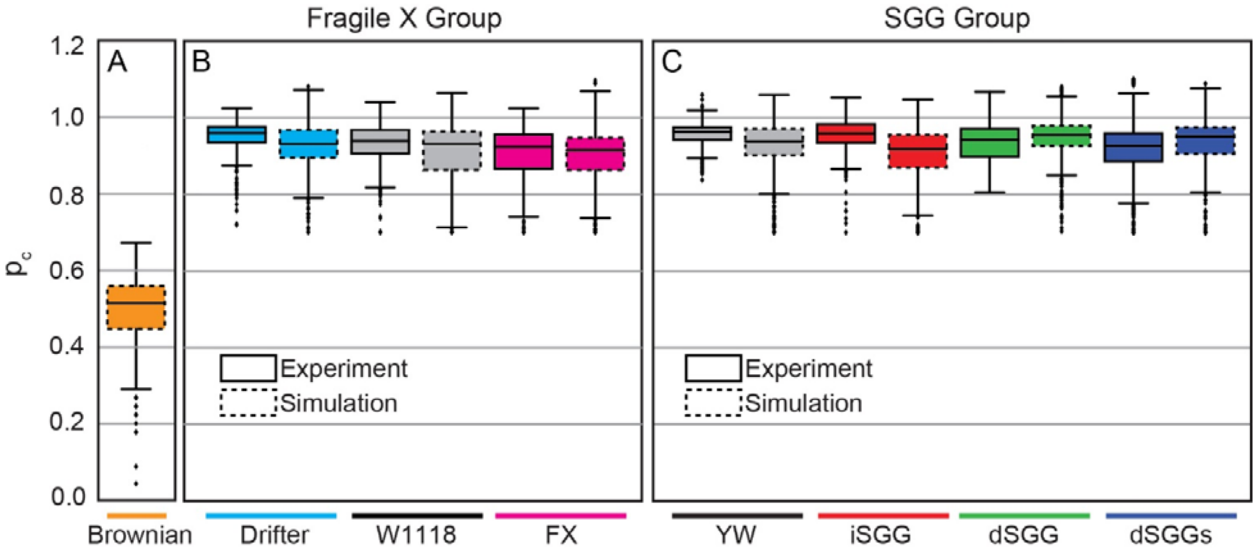

<u>Supplementary Figure 4</u>: Experimental and simulated trajectories stem from the same diffusive process as classified by a renormalization group classification algorithm. *The renormalization group classification was previously described by O'Malley at al.* [13]*. Briefly, a family of renormalization group operators, $R_{n,p}$, is defined, the elements of which map trajectories to rescaled trajectory $x \rightarrow R_{n,p}\, x = x'$. For values of p that are too large, $R_{n,p}$ flattens the trajectory into x = 0 as n gets larger, while for values of p that are too small, $R_{n,p}$ maps x to trajectories with increasing variance in their displacements as n gets larger. For an intermediate value p_c, however, $R_{n,pc}\, x = x$ as a distribution. Our implementation (inspired by Regner et al.* [14]*) compares the original trajectory x to $R_{n,p}\, x$ for a range of p using a Kuiper two-sample test, and keeps values of p which minimize the two-sample test. We use a continuous time renormalization group $R_{dt,p}$, to accommodate data which is sampled at unequal intervals, and which agrees with the $R_{n,p}$ for data sampled at equal intervals. As the Tukey box-plot in (A) demonstrates, our implementation recovers the $p_c = 0.5$ expected for Brownian motion*[13]*. For all larvae strains from the Fragile X (B) and the SGG (C) groups, $p_c \approx 0.95$ for both experimental and simulated trajectories. This supports the model that all of the strains are accurately captured by the bimodal persistent random walk used for the simulations as described in the main text.*

Supplementary Table 1: Fitting parameters of the survival probability analysis of flight lengths by segmenting the tracks

Larva type	Slope ($-\lambda$)	r^2	p value	N
w1118	-1.2602	0.9885	4.24E-293	263
Fragile X	-1.5979	0.98641	1.38E-205	191
Drifter	-0.90783	0.96629	3.83E-146	166
YW	-0.86042	0.94313	4.04E-162	211
Increased SGG(iSGG)	-1.2309	0.98021	3.73E-179	180
Decreased SGG(dSGG)	-1.1429	0.95247	3.27E-115	143
Decreased SGG (severe) (dSGGs)	-1.3337	0.95608	1.51E-74	91

Estimated values of the parameter λ of an exponential distribution $\lambda e^{\lambda x}$ from the fit to the survival probability functions. In addition, the correlation coefficient r^2 the p-value of the test statistic are given. N is the number of data points.

Supplementary Table 2: MLE parameters of the fits for the step length distribution of the smoothed tracks

Larva Type	Distribution	Aikaike weights	AICc	$-\ln(L)$
w1118	Weibull	100%	-4312.53	-2158.27
	Gamma	0%	-4241.13	-2122.57
Fragile X (FX)	Weibull	100%	-3015.11	-1509.56
	Gamma	0%	-2958.92	-1481.46
Drifter	Weibull	100%	-3768.2	-1886.11
	Gamma	0%	-3689.84	-1846.92
YW	Weibull	100%	-4543.19	-2273.6
	Gamma	0%	-4490.57	-2247.29
Increased SGG (iSGG)	Weibull	100%	-3170.03	-1587.02
	Gamma	0%	-3116.02	-1560.02
Decreased SGG (dSGG)	Weibull	100%	-2907.67	-1455.84
	Gamma	0%	-2867.76	-1435.89
Decreased SGG (severe)	Weibull	100%	-1613.05	-808.54
(dSGGs)	Gamma	0%	-1588.46	-796.24

Fits of the two most commonly used step length models for PRWs, Weibull and Gamma distributions, are compared for each strain. The negative log-likelihood values, $-\ln(L)$, are estimated with maximum likelihood estimation (MLE). The values of the corrected Akaike information criterion (AICc) and the Akaike weights are determined from these. The Weibull distribution was consistently found to best fit the step length distribution for all larva strains tested.

Supplementary Table 3: Parameters of the fitted Weibull functions for the step length distribution of the smoothed tracks

Larva type	Scale parameter a	Shape parameter b
w1118	0.087661	1.5008
Fragile X	0.084893	1.5078
Drifter	0.088952	1.5785
YW	0.091627	1.4346
Increased SGG (iSGG)	0.086266	1.4871
Decreased SGG (dSGG)	0.084846	1.5006
Decreased SGG (severe) (dSGGs)	0.084023	1.6226
Mean $\pm$ STD	0.08690 $\pm$ 0.0027	1.519$\pm$0.062

Best fit parameters of the Weibull distribution for the step lengths, estimated using MLE. The mean and standard deviation of these values was used in simulations, but care must be taken when studying other strains as their step length distribution could be altered by mutation.

Supplementary Table 4: MLE fitting parameters of the turning angle distributions between smoothed points

Larva type		w1118	FX	Drifter	YW	iSGG	dSGG	dSGGs
$-\log(L)$ (Gauss)		3896.97	2396.55	3501.54	4394.87	2662.53	2389.62	1129.8
$-\log(L)$ (Cauchy)		4407.15	2772.83	3822.36	4793.6	2992.18	2669.2	1216.2
$N_{\text{all data}}$		1300	883	1128	1428	948	848	452
$N_{\text{reorientation}}$		212	201	141	182	179	166	127
N_{crawling}		1088	682	987	1246	769	682	325
$N_{\text{crawling,truncated}}$		1040	640	962	1212	738	654	316
$-\log(L)\ /N$ (Gauss)		3.75	3.74	3.64	3.63	3.61	3.65	3.58
$-\log(L)\ /N$ (Cauchy)		4.05	4.07	3.87	3.85	3.89	3.91	3.74
Gaussian fitting params. [degree]	σ	10.26	10.23	9.22	9.09	8.92	9.35	8.64
	CI	0.44	0.56	0.41	0.36	0.46	0.51	0.67

The number of data points and MLE results of the fits of the angular distributions between smoothed points. The normal and Cauchy distributions, commonly used to fit the turning angle distributions, were tested. As Figure 3 shows, the distribution of turning angles after separating the reorientation events still contains a few outlying events, which could either indicate undetected reorientation events or be part of the long tail of a Cauchy distribution. For fits representing the first hypothesis we removed these outliers by applying Grubb's test to the turning angles of the active crawling phase. The normal distribution was fitted to the data points $N_{\text{crawling,tuncated}}$, while the long-tailed Cauchy distribution was fitted to the data points N_{crawling}. A comparison of the log-likelihood values can be done after normalizing it by the respective number of data points. The smaller value represents the better fit, which is the normal distribution for all strains.

Supplementary Table 5: Summary of the bimodal PRW parameters for all larvae strains

Larva type		w1118	FX	Drifter	YW	iSGG	dSGG	dSGGs	w1118 control
Frequency of RP [%]	Mean	16.31	22.76	12.50	12.75	18.88	19.58	28.10	16.02
	SEM sub.	1.34	3.21	0.70	1.53	1.40	3.09	3.15	0.90
Time per CP [s]	Mean	5.64	6.20	4.60	4.40	5.38	6.94	7.56	5.78
	STD	2.28	2.67	1.70	1.85	1.97	2.99	2.88	2.41
	SEM	0.16	0.19	0.14	0.14	0.15	0.23	0.26	0.22
Time per RP [s]	Mean	16.13	21.89	12.34	14.55	17.21	23.28	25.13	15.91
	STD	10.66	14.02	5.51	12.91	9.41	15.38	15.92	10.34
	SEM	0.73	0.99	0.46	0.96	0.70	1.19	1.41	0.96
Time per RP/ time per CP	Mean	2.86	3.53	2.68	3.31	3.20	3.35	3.33	2.75
	SEM prop.	0.15	0.19	0.13	0.24	0.16	0.21	0.22	0.20
Persistence length, l_p	Mean:	8.10	8.32	10.07	10.55	10.96	9.89	11.43	-
	STD:	0.72	0.93	1.02	0.78	0.92	1.13	1.78	-
	SEM:	0.14	0.18	0.19	0.14	0.16	0.21	0.32	-

All values that are used to characterize differences between the wild-type and mutant strains are summarized. All standard deviations (STD) and standard errors of the mean (SEM) were calculated in the usual way. ‘SEM sub.’ refers to standard errors of the means estimated by sub-sampling 12 tracks of each strain, calculating the respective parameter and repeating 1000 times. ‘SEM prop.' refers to error bars estimated by propagation of errors. RP: Reorientation points. CP: Crawling points.

Supplementary Table 6: Additional parameters of the bimodal PRW used for simulations

Larva type		w1118	FX	Drifter	YW	iSGG	dSGG	dSGGs
Gamma distribution parameters (time per RP [s])	k	4.7966	3.6019	6.5606	2.4862	4.6594	4.2287	3.7247
	CI(k)	0.8067	0.6148	1.3362	0.4369	0.8454	0.7914	0.7823
	Θ	3.3632	6.0774	1.8810	5.8522	3.6929	5.5045	6.7480
	CI(Θ)	0.5934	1.1058	0.3964	1.1279	0.7036	1.0871	1.5053
Gamma distribution parameters (time per CP [s])	k	6.2825	5.2914	7.0491	5.7128	7.3964	5.1316	6.6659
	CI(k)	0.4940	0.5177	0.5823	0.4199	0.6890	0.5017	0.9289
	Θ	0.8980	1.1722	0.6523	0.7704	0.7275	1.3527	1.1337
	CI(Θ)	0.0734	0.1200	0.0558	0.0591	0.0700	0.1386	0.1636
Gaussian parameters (turning angles after RP [degree])	σ	54.33	57.00	45.38	40.09	54.90	37.50	39.53
	CI(σ)	5.22	5.72	5.43	4.15	5.71	4.17	4.93

The time per reorientation point (RP) as well as per active crawling point (CP) were modeled as gamma distributions with shape parameter k and scale parameter Θ. The turning angle distribution after reorientation points was modeled as a Gaussian distribution with mean 0 and variance σ. All shown parameters and confidence intervals (CI) were estimated using MLE fitting.

References

1 Jakubowski, B. R., Longoria, R. A. & Shubeita, G. T. A high throughput and sensitive method correlates neuronal disorder genotypes to Drosophila larvae crawling phenotypes. *Fly* **6**, 303-308, doi:10.4161/fly.21582 (2012).

2 de Jager, M., Weissing, F. J., Herman, P. M., Nolet, B. A. & van de Koppel, J. Levy walks evolve through interaction between movement and environmental complexity. *Science* **332**, 1551-1553, doi:10.1126/science.1201187 (2011).

3 Humphries, N. E. *et al.* Environmental context explains Levy and Brownian movement patterns of marine predators. *Nature* **465**, 1066-1069 (2010).

4 Sims, D. W. *et al.* Scaling laws of marine predator search behaviour. *Nature* **451**, 1098-1102, doi:10.1038/nature06518 (2008).

5 Viswanathan, G. M. *et al.* Levy flight search patterns of wandering albatrosses. *Nature* **381**, 413-415 (1996).

6 Viswanathan, G. M. *et al.* Optimizing the success of random searches. *Nature* **401**, 911-914 (1999).

7 Edwards, A. M. Overturning conclusions of Levy flight movement patterns by fishing boats and foraging animals. *Ecology* **92**, 1247-1257 (2011).

8 Edwards, A. M., Freeman, M. P., Breed, G. A. & Jonsen, I. D. Incorrect likelihood methods were used to infer scaling laws of marine predator search behaviour. *PloS one* **7**, e45174 (2012).

9 Edwards, A. M. *et al.* Revisiting Levy flight search patterns of wandering albatrosses, bumblebees and deer. *Nature* **449**, 1044-1048 (2007).

10 Potdar, A. A., Jeon, J., Weaver, A. M., Quaranta, V. & Cummings, P. T. Human mammary epithelial cells exhibit a bimodal correlated random walk pattern. *PLoS One* **5**, e9636, doi:10.1371/journal.pone.0009636 (2010).

11 Sims, D. W., Righton, D. & Pitchford, J. W. Minimizing errors in identifying Levy flight behaviour of organisms. *J Anim Ecol* **76**, 222-229, doi:10.1111/j.1365-2656.2006.01208.x (2007).

12 Wilhelm, J. & Frey, E. Radial Distribution Function of Semiflexible Polymers. *Physical Review Letters* **77**, 2581-2584 (1996).

13 O'Malley, D. & Cushman, J. H. A Renormalization Group Classification of Nonstationary and/or Infinite Second Moment Diffusive Processes. *J Stat Phys* **146**, 989-1000, doi:10.1007/s10955-012-0448-3 (2012).

14 Regner, B. M., Tartakovsky, D. M. & Sejnowski, T. J. Identifying Transport Behavior of Single-Molecule Trajectories. *Biophys J* **107**, 2345-2351, doi:10.1016/j.bpj.2014.10.005 (2014).
